# Treatment of Migraine With Phytocannabinoids, the Involvement of Endocannabinoids in Migraine, and Potential Mechanisms of Action

**DOI:** 10.1155/prm/7181066

**Published:** 2025-07-01

**Authors:** Roger Gregory Biringer

**Affiliations:** College of Osteopathic Medicine, Lake Erie College of Osteopathic Medicine, 5000 Lakewood Ranch Blvd., Bradenton 34211, Florida, USA

**Keywords:** endocannabinoid, headache, migraine, nociception, phytocannabinoid

## Abstract

The American Migraine Foundation estimates that over 39 million Americans and over 1 billion people worldwide suffer from some form of migraine. Treatment of migraine generally falls into two categories: treatment of attacks once they have begun, and prophylactic prevention, including lifestyle changes. The use of phytocannabinoids to reduce both the frequency and severity of migraine is widely documented in scientific, grey, and popular literature. This review provides descriptions of both preclinical and clinical studies involving the treatment of migraines with phytocannabinoids as well as the involvement of endocannabinoids and endocannabinoid-like compounds in migraine pathology, including the receptors and associated mechanisms. Currently unanswered questions and areas for further exploration are discussed.

## 1. Introduction

The American Migraine Foundation (https://americanmigrainefoundation.org/) estimates that over 39 million Americans and over 1 billion people worldwide suffer from some form of migraine. Migraine headaches are typically unilateral but may be bilateral or front to back and can last from hours to days (reviews [1–3]). Migraines are often accompanied by conditions such as photophobia, phonophobia, and osmophobia, as well as nausea and vomiting. About a quarter of migraineurs experience an aura phase just prior to the headache pain that involves visual abnormalities such as seeing sparks or dots.

The generally accepted theory for migraine pathophysiology is that it is neurovascular in nature where vasodilation, vasoconstriction, and neuro-activation play important roles [3–6]. Headache typically begins with a vasospasm—a small vasoconstriction followed by a larger vasodilation—leading to the activation of the trigeminal vascular system, including the trigeminal nerve [7–13]. This, in turn, serves as a trigger for the release of calcitonin receptor gene-related peptide (CGRP), substance P, and vasoactive intestinal peptide from the trigeminal sensory C fibers, which leads to vasodilation of the meningeal vessels and neurogenic inflammation [14–16]. Currently, it is believed that the neurological component is the primary cause of migraines and the vascular component results from the neurological condition involving trigeminal nociceptive activation [3, 5, 6, 17]. In parallel with this, central sensitization, an increased responsiveness of nociceptors in the central nervous system (CNS), also plays a role in migraine pathology [18]. Here, an increasing intensity of peripheral sensitization leads to central sensitization and an amplification of pain.

Treatment of migraine generally falls into two categories, treatment of attacks once they have begun and prophylactic prevention, including lifestyle changes (reviews [19, 20]). The standby for specific treatment of acute migraine has been the triptan family of pharmaceuticals, alone or in combination with nonsteroidal anti-inflammatory drugs (NSAIDs). However, triptans are not effective in all patients or patients who are already in the state of hypersensitization. In response, new families of pharmaceuticals, such as 5HT1F-agonist Lasmiditan (ditan family) and the CGRP receptor blockers (gepants family), are currently being developed and tested [21]. NSAIDs alone are often used in the treatment of mild acute migraine. Prophylactic pharmaceutical treatments are in common use (e.g., propranolol) but must be individualized to the patient. More recently, onabutulinumtoxin A and CGRP monoclonal antibodies have been proven effective as prophylactic treatments, particularly for chronic migraine (CM) [21, 22]. Further complicating treatment is that many factors can trigger migraine, including changes in barometric pressure, temperature, hydration, sleep disturbance, missing meals, stress, and hormonal fluctuations [23–25] as well as patient-specific dietary triggers [26].

In addition to the pharmaceuticals noted above, phytocannabinoids have been used to treat and reduce the frequency of migraine. Both raw plant materials (i.e.*, Cannabis sativa*) *and extracts of plant materials have been shown to be effective in reducing both the frequency and severity of migraine headaches*. This review provides descriptions of both preclinical and clinical studies involving the treatment of migraine with phytocannabinoids and details the receptors and mechanisms shown to be involved in the treatment of migraine. The review goes on to explore how the known endogenous cannabinoids, the endocannabinoids, as well as endocannabinoid-like substances may be involved in migraine pathology.

## 2. Medical Marijuana and Phytocannabinoids

**Figure 1:**
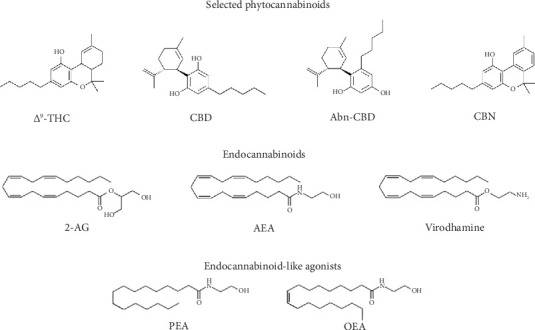
Structures for selected phytocannabinoids, endocannabinoids, and endocannabinoid-like agonists. Abbreviations: 2-AG, 2-arachidonylglycerol; Abn-CBD, abnormal cannabidiol; AEA, anandamide; CBD, cannabidiol; CBN, cannabinol; Δ^9^-THC, Δ^9^-tetrahydrocannabinol; OEA, oleoylethanolamide; PEA, palmitoylethanolamide.

The *Cannabis sativa* plant has been used for centuries for both medical and recreational uses. As of 2021, cannabis contains at least 554 compounds, of which 113 are cannabinoids, known as phytocannabinoids, from 11 different classes (reviews [27, 28]). Four of the most cited phytocannabinoids for both medical and recreational use are depicted in Figure 1. The relative concentrations of the various phytocannabinoids are dependent on the environment in which the plant was raised and the strain of cannabis itself. The cannabinoid most noted for its psychoactive effect is Δ^9^-tetrahydrocannabinol (Δ^9^-THC), but it is not the only known psychoactive constituent, albeit the most potent. For example, Δ^8^-tetrahydrocannabinol is also known to be psychoactive but to a much lesser degree. Other constituents commonly noted in the literature include cannabidiol (CBD), abnormal cannabidiol, (ABN-CBD), and cannabinol (CBN) are not considered to be psychoactive at all.

## 3. Results

### 3.1. Cannabinoid Use for the Treatment of Acute Migraine: Clinical and Preclinical Studies

Cannabinoid use for medical purposes has been documented by many over the years (reviews: [29–33]). However, the lack of significant numbers of clinical studies makes the conclusions tenuous, and this fact is at the heart of the rationale for this review, which is to promote more clinical studies on cannabinoid treatment for acute migraines. Only one recent clinical study addresses cannabinoid use for the treatment of acute migraines in a randomized, double-blind, placebo-controlled study. Here, 92 individuals inhaled vaporized Δ^9^-THC and CBD alone or in combination, and the efficacy of treatment was examined [34]. Individually, Δ^9^-THC and CBD were less effective than in combination. A combination of 6% Δ^9^-THC and 11% CBD was found to be superior to placebo in terms of pain relief, pain freedom, and most bothersome symptoms at 2 h postvaporization, with sustained benefits at 24 and 48 h and produced less euphoria and cognitive impairment than Δ^9^-THC. Interestingly, CBD was found not to be superior to placebo in any manner examined at 2 h, and Δ^9^-THC alone was superior to placebo only in terms of pain relief at 2 h where it was roughly equivalent to the Δ^9^-THC/CBD mixture. Interestingly, in a preclinical study, injection of CBD was shown to significantly reduce hyperalgesia in a nitroglycerin (NTG) induced rat migraine model [35]. A second, smaller clinical cohort study of 32 chronic migraineurs who were not responding to other treatments, or if such treatments were contraindicated [36]. CM patients previously treated at the University of Modena clinic were prescribed daily oral doses of one of three different mixtures of CBD and Δ^9^-THC available as off-label commercial preparations. Participants were asked to track their migraine experience prior to treatment as a baseline and then at 3 and 6 months after starting the treatment. The results show that regardless of the treatment formulations, the number of migraine days did not change significantly from baseline. However, the pain intensity, acute medication consumption, and the number of days per month such acute medications were taken decreased significantly over the course of the study. Although this study gives a promising result for migraine pain reduction through cannabinoid prophylaxis, the small number of patients involved is a major limitation to its generalization, but at the same time, it suggests that more and larger clinical studies are required to confirm or dismiss these findings.

Most studies examining the effect of cannabinoids for the treatment of migraine rely on self-reported surveys without controls, and this fact may lend some suspicion to their validity. Concerns over self-reporting have been reviewed elsewhere [37–39] and will not be addressed here, but the results presented here are those published and are left to the reader for consideration. However, a recent study explored this validity question by examining the reasons for the use of medical cannabis and the perceived effects, which it is appropriate to note here. Although they examined several other pain-related conditions, such as post-traumatic stress disorder (PTSD), chronic pain, and depression, relief of migraine/headache pain was represented by 21.2% of the 632 participants [40]. Of this group, 78.9% reported improvement in their condition by using medical cannabis. In addition to efficacy, they examined the conditions that individuals reported as the main reason for medical marijuana use and found a 70%–90% correlation for all conditions. Although this does not by any means validate the use of self-reporting, it does suggest that users in this study had a purpose in mind beyond any associated euphoric effects.

One recent study presents self-reported cannabis use from a 2018 survey of 27,169 respondents in both the United States and Canada [41]. Of those reporting medical use (27% of total respondents), 35% reported using cannabis for treatment of headache or migraine. However, the frequency of use and efficacy of treatment were not determined. Similarly, a survey of 2032 medical cannabis patients revealed that 24.9% treated headaches with medical cannabis [42]. Utilizing the IDMigraine questionnaire, 88% of these patients were treating probable migraine. A more directed survey was reported for 200 patients presenting with headaches at a tertiary headache clinic (Calgary, Alberta, Canada) [43]. In this study, 60% of patients indicated improvements in the severity of headaches, and 25% of the patients indicated that cannabinoid use reduced the frequency of headaches. Of these, 44.9% were diagnosed with CM and 11.2% with episodic migraine. The frequency of cannabinoid usage for headache treatment varied with about 45% indicating usage once a month or less. Similarly, in a recent literature review of clinical findings for 1980 medical cannabis users, cannabis was reported to abort migraine in 11.6% of users and reduced the frequency of migraine [44]. More specifically, in a survey of 589 adult cannabis users, 27.3% reported experiencing migraines, 76.4% of these (*n* = 123) endorsed using cannabis to relieve migraine, and 69.9% found cannabis to be superior to other noncannabinoid products (e.g., triptans, over-the-counter medications) for relief from migraine [45]. The most recent reported survey found involves patients from a tertiary headache center where 55.7% of the 1373 reporting indicated cannabis-based product use over the past 3 years [46]. The most common forms of delivery of the cannabis-based product were inhaled products and edibles. Of those reporting cannabis use, the majority experienced improvement in migraine headache including intensity (78.1%), duration (73.4%), frequency (62.4%), and nausea (56.3%). Close to half of these respondents (48.9%) found that cannabis-based product use reduced the need for other migraine medications, and some (14.5%) reported that they were able to eliminate other medications.

Most of the studies described above do not specifically address the efficacy with respect to the type of cannabis used to treat migraine. One study that does address this utilized data from the Strainpoint medical cannabis app, a software allowing individuals to track symptoms before and after cannabis use as well as the strains and dosages utilized [47]. Of the 12,293 sessions recorded, 60.5% referred to the treatment of migraine. The findings indicate that men reported slightly larger reductions in headaches than women and that inhaled cannabis reduced the severity of migraine by 50%. However, cannabis concentrates were more effective than smoking cannabis flowers, but the reduction in severity was not reported. Lastly, the effectiveness of cannabis treatment of migraine reduces with time, suggesting that a tolerance may develop with use.

The studies discussed here suggest that cannabinoid treatment in some form may be useful in alleviating migraine pain. However, more clinical research is necessary to examine various cannabinoid cocktails as well as delivery methods to determine which, if any, are the most effective for relieving all migraine-associated symptoms.

### 3.2. Clinical and Preclinical Studies Supporting the Involvement of Endocannabinoids in Migraine

Although the clinical studies outlined above require significant further study under more controlled conditions, phytocannabinoids appear to reduce the severity and frequency of migraines in some but not all users. The efficacy of phytocannabinoids, combinations thereof, and delivery methods remain undetermined. However, the results raise the question of how endocannabinoids fit into migraine pathology.

The classic endocannabinoids known to be cannabinoid receptor (both CB1 and CB2) agonists are the arachidonic derivatives anandamide (AEA), 2-arachidonic glycerol (2-AG), and the more recently discovered and lesser-known virodhamine (Figure 1) [48, 49]. There are, however, endocannabinoid-like compounds such as palmitoylethanolamide (PEA) and oleoylethanolamide (OEA) that are poor agonists for the cannabinoid receptors CB1 and CB2 but are very potent agonists for the orphan receptor GPR55, claimed by some to be the third cannabinoid receptor [48]. It is also noteworthy that both PEA and OEA are potent agonists for the peroxisome proliferator-activated receptor-α (PPARα) and peroxisome proliferator-activated receptor-γ (PPARγ), receptors that are associated with analgesic and nociceptive properties [50, 51]. Activation of both PPARα and PPARγ by AEA and 2-AG has also been reported [51, 52].

There is significant evidence that a dysfunctional endocannabinoid system has a major impact on migraine pathology. In particular, a deficiency in the maintenance of appropriate endocannabinoid concentrations (clinical endocannabinoid deficiency) has been shown to be involved in predisposing individuals to migraine attacks [53, 54]. Direct support for this was presented in a study of AEA levels in cerebrospinal fluid (CSF) obtained from 15 chronic migraineurs and 20 controls where it was found that AEA levels in the chronic migraineurs were 45% lower than observed for controls [55]. Similarly, salivary endocannabinoid levels were monitored for individuals with trigeminal neuralgia (TN) (*n* = 7), migraine (*n* = 5), and tension-type headache (TTH) (*n* = 11) [56]. The results showed that 2-AG levels were significantly lower in TN and TTH patients and that PEA levels were significantly lower in the migraine group. In contrast, a study involving 26 healthy women and 38 female migraineurs showed no significant differences in blood AEA or other N-acylethanolamine concentrations between the two groups [57]. In a third clinical study involving CM patients (*n* = 20) and controls (*n* = 20), platelet levels of both AEA and 2-AG were measured and found to be significantly lower in CM patients than controls, also indicating a dysfunction of the endocannabinoid system [58]. These data strongly suggest that reduced levels of endocannabinoids and related compounds promote the onset of migraine and other headache pathologies. Lastly, another clinical study involving 80 migraine patients, the effect of oral doses of PEA (Levagen+) (40 patients) on the resolution of migraines and reduction of pain intensity when given at the onset of migraine symptoms was compared to those (40 patients) taking oral doses of placebo at the onset of migraine [59]. The results showed that administration of PEA resolved significantly more headaches and reduced the pain levels in patients than those receiving placebo. These results not only support the dysfunctional endocannabinoid theory but also suggest that PEA is a safe and effective pharmaceutical for mitigating migraine. Similar studies utilizing OEA have not been reported to date.

A reduction in endocannabinoid levels could be due to a reduced rate of synthesis or enhanced rate of degradation. Although there are several synthetic and degradation routes for AEA as well as for PEA and OEA, the main synthesis enzyme for all of these is N-acyl-phosphatidylethanolamine-hydrolyzing phospholipase D and the main degradation enzyme is fatty-acid amide hydrolase isoforms 1 and 2 (FAAH) [60]. Similarly, there are multiple synthetic and degradation routes for 2-AG where the synthesis is primarily attributable to diacylglycerol lipases (DAGL) and the degradation is primarily attributable to monoacylglycerol lipase (MAGL) [61]. Lastly, enhanced membrane transport (EMT) activity can result in the sequestration or transport of endocannabinoids to other locations, resulting in reduced localized concentrations (review [62]). In one study involving 36 migraineurs without aura, 33 episodic tension-type headache patients (ETTH), and 36 controls, FAAH activity, EMT activity, and cannabinoid (CB) receptor levels were measured in isolated platelets [63]. The rationale for the utilization of platelets as a model for neuromodulator dysfunction is discussed elsewhere [58]. Platelet CB levels did not differ significantly between the three groups of patients. In addition, the results showed no difference in the activity levels for either FAAH or EMT in the male participants. For females, however, these activity levels were significantly higher than in males. Further, activity levels for female ETTH patients were the same as female controls, but both EMT and FAAH activity levels were significantly higher in migraine patients than female controls. The enhanced activities in these patients necessarily lead to reduced AEA levels. The authors suggest that this might be one of the causes for the high prevalence of migraine in females.

In a related study, platelet levels of both EMT and FAAH were measured for CM patients, (*n* = 21), episodic migraineurs (EM) (*n* = 28), and controls (*n* = 23) [64]. The results also show that EMT and FAAH activity levels for EM are higher in females than controls and that male levels are the same as controls. Interestingly, for both male and female CM patients, both EMT and FAAH activities were significantly lower than controls with male patients exhibiting lower activities than females. The authors suggest that this represents a compensatory mechanism to maintain AEA levels as high as possible in CM patients. Similarly, in a preclinical study utilizing a medication overuse episodic headache model in rats, female rats had a higher sensitivity to inhibition of the 2-AG synthesis enzyme DAGLα [65].

Further support for endocannabinoid system dysfunction is found in several preclinical studies. Utilizing a NTG rat migraine model, systemic administration of the peripherally restricted FAAH inhibitor URB937 served to block NTG-induced hyperalgesia [66], supporting the involvement of AEA in the mitigation of migraine pain. In a later study utilizing the same model, administration of the FAAH inhibitor URB597 before NTG administration significantly elevated levels of both AEA and PEA in the medulla, cervical spinal cord, and trigeminal ganglia, peripheral neural structures known to be involved in migraine pathology [67]. The elevated levels of AEA and PEA occurred simultaneously with a reduction in rat nocifensive behavior following formalin injection, an indication of an antinociceptive response to the formalin insult [66, 67]. When URB597 was administered 3 h after NTG administration, however, levels of AEA and PEA were not elevated in the trigeminal ganglia, PEA was not elevated in the cervical spinal cord, and rat nocifensive behavior was unaffected. The authors conclude that inhibition of FAAH may be useful as a preventative treatment in migraine but not an abortive one. In a similar study, pretreatment of rats with AEA prior to NTG injection also showed a significant decrease in nociceptive behavior [68]. Further support is found in a study involving FAAH and MAGL knockout mice using an NTG-induced mouse migraine model [69]. The results show that both NTG-induced mechanical allodynia and activation of the trigeminal nucleus was completely abolished in the FAAH knockout mice, supporting AEA as an antinociceptive agent. The NTG-induced pain reduction was not observed for the MAGL knockout mice, indicating that 2-AG is not involved. Additional results supporting AEA as an antinociceptive agent were obtained utilizing JZL195, a dual inhibitor of both FAAH and MAGL, on NTG-induced hyperalgesia [70]. The results showed a significant reduction in NTG-induced hyperalgesia as well as a reduction in behavior associated with pain.

### 3.3. Endocannabinoid/Cannabinoid Receptors

The clinical and preclinical studies discussed above strongly support the involvement of endocannabinoids in migraine pathology. This section presents preclinical data supporting the involvement of specific receptors.

Endocannabinoids and phytocannabinoids are known agonists for both type 1 (CB1) and type 2 (CB2) antinociceptive cannabinoid receptors. There are also reports that the orphan G-protein receptor GPR55 is also a target for endocannabinoids [71]; however, the inconsistency of results for this receptor makes this relationship rather controversial but nevertheless something to consider (reviews [48, 72]). CB1 receptors are expressed primarily in the CNS, but are also found in moderate amounts in adipose, female, lymphoid, and endocrine tissues, and in lower amounts in other tissues [73, 74]. The CB2 receptor is expressed primarily in leukocytes [72] but is also found in the brainstem [75] and in other tissues in low amounts. In the CNS, CB2 is primarily located in microglia but is also found in neurons [76, 77].

Both CB1 and CB2 are G-protein receptors and are known to signal through Gα_i/o_ resulting in inhibition of adenylate cyclase and activation of mitogen-activated protein kinase (MAPK) (reviews [75, 78–81]). CB1 has also been shown to inhibit voltage-gated Ca^2+^ channels and activate K^+^ currents, events known to reduce neuronal signaling, while reducing the release of nitric oxide (NO) via neuronal NO synthase (NOS), thus reducing vasodilation and migraine pain [80]. In contrast, CB1 and CB2 have also been shown to signal through the Gα_s_ pathway under certain conditions, leading to the activation of adenylate cyclase, and ultimately leading to the subsequent activation of specific kinases [77, 80–82]. In neurons, the primary effect of CB stimulation is the inhibition of the presynaptic release of neurotransmitters [77, 80], clearly supporting involvement in migraine pain mitigation as the clinical studies suggest. However, there are reports that stimulation of spinal CB1 receptors can be pronociceptive [83, 84].

GPR55 is expressed in high amounts in the brain [73], but its potential involvement in migraine pathology is complex and far from being understood. One complicating factor is the fact that different GPR55 agonists produce different outcomes. For example, the GPR55 agonist lysophosphatidyl inositol produces pronociceptive responses in rats [85, 86] by increasing intracellular calcium, a phenomenon also exhibited by Δ^9^-THC and AEA [87]. In contrast, the GPR55 agonists 2-AG, CBD, virodhamine, abn-CBD, and PEA were shown not to increase intracellular calcium [88], suggesting that no enhancement of excitatory neuronal signaling occurs with these agonists. In fact, CBD has been shown to antagonize THC-GPR55 interactions by mitigating the pronociceptive effect of Δ^9^-THC on this receptor [87]. A second complication is that GPR55 forms heteromers with both CB1 and CB2, resulting in modulation of CB or GPR55 signaling, the result of which depends on the CB-GPR55 heteromer [89]. Clearly, the effect of phytocannabinoids and endocannabinoids on migraine through GPR55 activation is dependent on the cannabinoid employed and if GPR55 is present in a heteromer with CB receptors.

It also has been established that both AEA and 2-AG (as well as PEA and OEA) are agonists for PPARα and PPARγ [51]. Furthermore, the phytocannabinoids Δ^9^-THC and CBD are also PPARα agonists [51]. The data for Δ^9^-THC and PPARγ are mixed [51]. PPARs form a heterodimer with the retinoid X receptor and, when activated with an appropriate ligand, serve as a transcription factor modulating several metabolic processes such as energy regulation, cell differentiation, and inflammation (reviews [90, 91]). PPARs are found in all tissues, including the brain where both PPARα and PPARγ are expressed in inhibitory neurons to a greater degree than excitatory neurons [74]. Activated PPARα and PPARγ are known to inhibit the activation of inflammatory gene expression and can also interfere with pro-inflammatory signaling pathways [91, 92]. There is also considerable support for the involvement of PPARα and PPARγ in the reduction of neuropathic pain [93–97].

In contrast to the antinociceptive effects discussed above, both AEA and 2-AG are known agonists for pronociceptive, heat, and capsaicin-mediated transient receptor potential cation channel subfamily Vanilloid member 1 TRPV1 [98, 99], suggesting a possible role in initiating migraine attacks, in clear opposition to the effects on the cannabinoid receptors [100–102]. Conversely, CBD is a known TRPV1 antagonist and desensitizes TRPV1 signaling, suggesting that it reduces pain sensitivity [103]. TRPV1 is a polymodal cation channel with a 10-fold higher preference for Ca^2+^ over other cations (reviews [104, 105]). This receptor is involved in a variety of nociceptive functions, including sensitivity to heat, pH, osmolality, and ischemia, as well as mediation of pain and inflammation. TRPV1 is expressed throughout the human CNS in roughly equal proportions in all areas of the brain [73]. Subcellularly, TRPV1 is found on postsynaptic dendritic spines of neurons [106, 107] and astroglia [107]. Activation of TRPV1 expressed on dural nerve fibers originating from the trigeminal ganglion results in the release of CGRP [108–110] from postsynaptic neurons, which serves to initiate a cAMP/PKA signaling pathway. This leads to the activation of NOS, resulting in the production and release of the vasodilatory signaling NO, causing subsequent vasodilation, that would serve to exacerbate migraine pain rather than relieve it [101, 111]. A potential rationale for the dichotomy of the action of endocannabinoids in migraine pathology is found in preclinical studies discussed below.

### 3.4. Clinical/Preclinical Support for Receptor-Based Mechanisms

Both CB1 and CB2 are activated by both endocannabinoids and some phytocannabinoids with efficacy dependent on both the agonist and the receptor [48]. In general, the EC_50_ values for endocannabinoids and both receptors show AEA < 2-AG < VH. For the phytocannabinoids, Δ^9^-THC is by far the strongest agonist, whereas CBN binding is orders of magnitude poorer, and CBD and abn-CBD have not been shown to activate either receptor. The reports published to date focus on CB1 as the primary cannabinoid receptor involved in migraine pathology. The fact that CB1 is primarily expressed in the CNS and in all regions, whereas CB2 is only minimally expressed in the brainstem, is more than suggestive of a greater role for CB1 in migraine [74, 75]. A clinical study involving female migraineurs (*n* = 20) and healthy controls (*n* = 18) utilized positron emission tomography which showed increased binding of the CB1 receptor agonist [^18^F]MK-9470 for migraine patients in the interictal state compared to healthy controls and was particularly evident in the cingulo-frontal cortex and limbic system, both known to be involved in pain modulation [112]. Utilizing an NTG rat migraine model, the CB1-selective antagonist rimonabant completely restored hyperalgesia under conditions where AEA concentrations were raised either by inhibition of the degradation enzyme FAAH or in FAAH knockout rats, indicating that CB1 alone is responsible for the analgesic effect of AEA [69]. CB2-specific antagonists were not examined, and thus, any contribution provided by this receptor cannot be entirely ruled out. An earlier preclinical study demonstrated that the CB1 antagonist SR441716 reversed trigeminal firing resulting from electrical stimulation of dura mater, whereas the CB2 receptor antagonist AM630 showed no effect, indicating that the response is likely to be CB1-mediated [113]. It is well established that vasoactive and neuroinflammatory peptides such as CGRP and substance P are released into the meninges vasculature and ultimately lead to migraine pain [15, 114–116]. Utilizing the release of CGRP from trigeminal ganglia as a measure of enhancement of neurogenic inflammation, intraperitoneal administration of methanandamide, a CB receptor agonist, was found to reduce CGRP release into plasma in a rat migraine model [117]. The addition of the CB1-specific antagonist rimonabant was found to attenuate the effects of methanandamide, but the CB2-specific antagonist SR144528 did not, suggesting again that CB1 receptors and not CB2 are involved in the modulation of CGRP release. In a related study utilizing a similar rat model, AEA was shown to inhibit both NO-induced and CGRP-induced vasodilation [118]. The NO-induced effect was reversed by the addition of the CB1-specific agonist AM251 but had no effect on CGRP-induced vasodilation unlike the effect shown by methanamide. These results suggest that CB1 is involved in the NO pathway, but in contrast, it indicates that the CGRP path is modulated by AEA through another receptor. The differences between these two studies remain unresolved.

Although there is significant support for a major role for CB1 in migraine pathology, there are reports suggesting a role for CB2 as well. Selective activation of CB2 receptors by AM1241 in a NTG-induced rat migraine model showed a significant analgesic effect [119], and thus, CB2 should not be neglected in a discussion of migraine pathology. This result is not unexpected, as activation of CB2 receptors has been shown to be involved in the attenuation of both neuropathic and inflammatory pain [120, 121]. Further support for the roles of both CB1 and CB2 in migraine pathology is found in a study utilizing a rat NTG-migraine model in both acute and chronic settings [122]. In the acute and chronic models, NTG-induced sensitivity to pain was reduced by the administration of the nonselective CB receptor agonist WIN 55,212-2. In the acute model, only the CB1 antagonist AM251 abrogated the protective effect. However, in the chronic model, utilization of WIN 55,212-2 in the presence and absence of AM251 attenuated NTG-induced hyperalgesia through both CB1 and CB2 with CB2 exhibiting the most prominent role. This suggests that an increase in CB2 expression is a protective response to CM. Further, considering the lack of CB2 control of vasodilation, CB2 appears instead to be involved in antinociception activity.

Contrary to the results discussed above, there are reports that CB1 can act in a pronociceptive manner. Stimulation of CB1 in the dorsal horn of rats has been shown to decrease the release of the neurotransmitter gamma-aminobutyric acid, thus facilitating the release of substance P, resulting in a pronociceptive effect [83]. Interestingly, like opioids, repeated stimulation with CB1 agonists has been shown to elicit abnormal pain and increased sensitivity to non-noxious stimuli [84]. This is a particularly important result to consider when addressing cannabinoid use for chronic conditions or overuse of cannabinoids for prophylaxis.

Currently, there are no clinical or preclinical studies that directly implicate GPR55 in migraine pathology. However, there are a few studies that suggest its involvement. As noted above, the phytocannabinoid CBD is a major component of marijuana smoke and oral cannabinoid preparations, which have been shown to be effective in reducing the severity of migraine pain [36, 47]. However, CBD is known not to be an effective agonist/antagonist for either CB1 or CB2 [71] but a strong antagonist for GPR55 that causes an increase in inhibitory neuronal activity [123]. Although this study's measure of antagonist activity was a reduction in the seizure activity in mice, the resulting reduction in neutral transmission might well be applicable to migraine [87]. PEA is a known endocannabinoid-like compound that has been shown to reduce both the pain intensity and migraine frequency in pediatric migraine patients [124]. Furthermore, significantly lower PEA levels were found in the saliva of migraineurs than controls [56]. These studies suggest that PEA can mitigate migraine severity and frequency, and low levels predispose individuals to migraine attacks. The fact that PEA is a very strong agonist for GPR55 and does not bind to either CB1 or CB2 [71] suggests that its antimigraine activity might well involve the GPR55 receptor. However, PEA is also a strong agonist for PPARα, which is known for its involvement in anti-inflammatory mechanisms [125], and the PEA-driven effect might also be through this pathway. There are also known indirect effects through the PEA inhibition of FAAH, which leads to increased amounts of AEA, potentially leading to the activation of the CB receptors [124].

Only one clinical study on the relationship between PPARs and migraine has been reported [126]. In this study, blood levels of PPARα, PPARβ/*δ*, and (*n* = 98), interictal state (*n* = 129) as well as in healthy controls (*n* = 100). The results showed that both PPARα and PPARβ/*δ* were significantly higher for migraineurs in the ictal state than in the interictal state or for controls which were similar. In contrast, PPARγ levels were significantly lower in the ictal migraineurs compared to interictal migraineurs or controls which were similar. The authors suggest that PPARα and PPARβ/*δ* serve in a protective role during migraine and that the reduction of PPARγ accelerates migraine. This compares well with the work of others which have shown that both PPARα and PPARβ/*δ* are involved in anti-inflammatory effects and PPARγ has been shown to be a negative regulator of inflammatory genes [93, 127].

There is significant support for both AEA and 2-AG, acting as agonists for the TRPV1 receptor, ultimately leading to the release of CGRP and significant vasodilation that may lead to migraine [98, 99]. This is in direct contrast to their vasoconstrictive action on the CB1 receptor, a dichotomy that may serve as a concentration-sensitive modulator of the endocannabinoid activity in migraine pathology [99–102]. Utilizing isolated rat and guinea pig arteries, AEA has been shown to induce vasodilation through the release of CGRP [128]. Both endogenous (2-AG and PEA) and synthetic CB agonists (HU 210, WIN 55,212-2, and CP 55,940) did not mimic this effect, indicating that another receptor other than one of the CB receptors is involved. Patch clamp experiments with cloned TRPV1-containing HEK-293 cells confirmed that AEA induces capsazepine-sensitive currents in both whole cells and isolated membrane patches, indicating that AEA acts through TRPV1/CGRP to induce vasodilation. In contrast, a more recent manuscript reveals that endogenous 2-AG (as well as 1-AG) does induce vasodilation through TRPV1 in rat mesenteric arteries and that PEA enhances the effect [98]. Support for AEA-inducing TRPV1/CGRP vasodilation is provided in a later study utilizing intravital microscopy to examine the effect of AEA on vasodilation of rat dural vessels [129]. Here, AEA was shown to induce vasodilation, and both TRPV1 antagonists capsazepine and CGRP_8-37_ reduce the effect, indicating that AEA acts through the TRPV1 receptor. Further, pretreating rats with the CB1 receptor antagonist had no effect on AEA-induced vasodilation, indicating the CB1 receptor was unable to modulate the activity and that its actions are independent of CB1. It has been suggested that CB1 independence may depend on the colocalization of the two receptors and that in this situation, AEA likely activates the CB1 receptor at low concentrations to produce vasoconstriction and promotes vasodilation at higher AEA concentrations through the TRPV1 receptor [99]. The latter hypothesis is supported by a study involving cultured rat primary sensory neurons which express both CB1 and TRPV1 [130]. Here, it was found that at low concentrations (< 1 μM), AEA inhibited the release of CGRP by stimulating CB1 receptors but stimulated TRPV1-mediated release of CGRP at higher concentrations (> 1 µM). Support for a similar mechanism in humans is found in the relative EC_50_ values obtained for endocannabinoid stimulation of human CB1 and TRPV1 where the values for AEA and 2-AG stimulation of CB1 [71] are significantly lower than those found for TRPV1 [131]. Further, the expression levels for CB1 are significantly higher than TRPV1 in all areas of the brain [73], also supporting the dominance of AEA inhibition of CGRP release.

## 4. Conclusion

The clinical studies published to date strongly suggest that phytocannabinoids are useful for mitigating migraine pain and for migraine prophylaxis. Further, studies show the potential for endocannabinoid and endocannabinoid-like compounds in migraine treatment. It is also clear that new, additional clinical trials utilizing specific phytocannabinoids and combinations of phytocannabinoids are necessary to determine the best course for migraine treatment. Further, additional clinical studies involving endocannabinoid and endocannabinoid-like compounds for the treatment of migraine would provide insight into alternative pharmaceuticals for the treatment of migraine. Lastly, more studies involving the inhibition of endocannabinoid degradation enzymes may very well provide support for another avenue for migraine treatment.

Studies reveal that both CB1 and CB2 are involved in migraine pathology with CB1 primarily involved with the vasodilatory aspects and CB2 with nociception. The involvement of GPR55 in migraine pathology is still unclear due primarily to its agonist dependence and the dearth of preclinical studies. Further work in this area would be most useful. TRPV1 working in concert with CB1 may represent an agonist concentration-dependent regulation of vasodilation. PPARα and PPARβ/*δ* appear to be involved in migraine resolution through their anti-inflammatory activities and PPARγ is involved in migraine onset. How these PPARs work in tandem remains unresolved and begs for further study.

## Data Availability

All data used for this manuscript are published in the literature.
